# Young Adults’ Perception of Forests Using Landscape-Image-Sketching Technique: A Case Study of Changsha, Central China

**DOI:** 10.3390/ijerph20042986

**Published:** 2023-02-08

**Authors:** Fen Luo, Chen Wang, Haiqian Lei, Zhijun Xiao

**Affiliations:** College of Tourism, Central South University of Forestry & Technology, Changsha 410004, China

**Keywords:** forest perception, landscape image, conceptual model, Central China

## Abstract

The forest-landscape image is the bridge for communication between human beings and the forest. The aim of this paper is to construct the landscape-image conceptual model from the personal perception of the forest, with what people are looking at and how they are viewing themselves as a part of the forest. This research constructed a forest-landscape image by young adults by utilizing the landscape-image-sketching technique and selecting 140 young adults who had lived in Changsha, Central China for ten years, using convenience sampling, during April and May 2018. The results demonstrated that the forest was considered as the people’s life world, as rural scenery around the respondents’ homes, instead of the perception of the objective forest, an important habitat for animals and a limited resource supplier for human living. In fact, the natural values of the forest, such as the ecological and aesthetic values, received more attention than the social ones of the forest, including the life, production, and cultural values. Finally, it is important to raise the public’s awareness of the objective entity of the forest and to guide the variety of experiences for the respondents in the forest.

## 1. Introduction

The utilization of the forest has been transformed into a strategic supply space for the ecological public goods of the forest [1]. Forest recreation is becoming a pillar industry for forestry in many countries, in line with timber harvesting and the sub-forestry economy. According to the national-forest-service programs, the United States produced USD 130.7 billion in extra value, or approximately 2% of GDP, with forest recreation accounting for almost 75% of the total output value produced in 2000, mainly in recreation and sports. Under the guidance of the forestry-development theory that “lucid waters and lush mountains are invaluable assets”, since 2012, studies in China on public perception of the forest have benefited the development of new forest-recreation products to meet the new needs of the people [2]. Previous studies demonstrated that the ecological environment played an important role in shaping the public’s happiness.

Since the introduction of the topic “forests in development—a vital balance” at the 13th World Forestry Congress in 2009, studies on individual perception have become one of the hot spots of the service functions of the forest ecological system [3,4,5,6,7,8]. These studies focused on public perceptions of the physical features of forest landscapes and the public perception of information [9,10]. Most of the published studies in China, however, focused on the natural ecological attributes of forests [11,12], and neglected the understanding of forests from the interaction between human beings and the forest [13].

A variety of methods for researching the public perceptions of and preferences regarding the forest landscape were applied in previous studies [7,14,15], such as the photo-grading method [16], the semantic-differential scale [17], the Delphi method [18], on-site interview [19], and multi-dimensional cognition and value-evaluation models [20]. In China, some research methods, such as the analytic hierarchy process (AHP) [21], the analytic network process (ANP) [22], and the scenic-beauty-estimation (SEB) method [23], and so on, were usually used to evaluate the forest aesthetic values [12], the forest landscape quality [24], and the multi-properties of the forest [2].

Recently, the visual-stimuli method was usually adopted to directly measure the individual perception of the forest landscape [7], concentrating on individual interpretations of the perceived forest information [25,26]. However, a gap was shown to exist between the employment of the visual stimulus for the assessment of individual perception of the forest landscape and those utilizing on-site forest-landscape experiences in these studies in Europe and the United States [3,27]. In addition, some new methods have become increasingly important, because they could effectively capture and understand the subjective perceptions and preferences of human beings for landscape [7,28]. The landscape-image-sketching technique in an Eastern context has rarely been investigated [3]. The landscape-image-sketching technique was thought of as an effective method for capturing human perceptions of forest landscapes [16]. This method involves a brief landscape sketch, keywords for the sketch, and concise oral descriptions of the landscape sketch by the respondents, which could reflect the respondents’ perceptions of the forest landscape.

The young adults (18–25 years old) have quite stable values and personalities, which could substantially affect their perception and attitude to the forest [29]. Meanwhile, they had strong expectations of fostering a relationship between their own children and the forest in similar ways as they themselves had acquired [30]. There is a tension between Chinese youth’s increased exposure to both Western individualism and traditional Confucian cultural values. Consequently, the aim of this research is to construct a forest-landscape-image model for the young adults in a Chinese context. To achieve our aim, ‘forest’ referred to a subjectively exhibited image of the forest in the respondents’ minds, in this research. This research was driven by two key objectives. Firstly, this research aimed to understand the Chinese cognition of a forest in terms of what people look at, and how they view themselves as a part of a forest. Secondly, this research aimed to construct a conceptual-image model of the interaction between the young adults and the forest, in a Chinese context. The contribution of this research was in the exploration of a conceptual-image model of the forest for young adults in a Chinese context.

## 2. Materials and Methods

**Figure 1:**
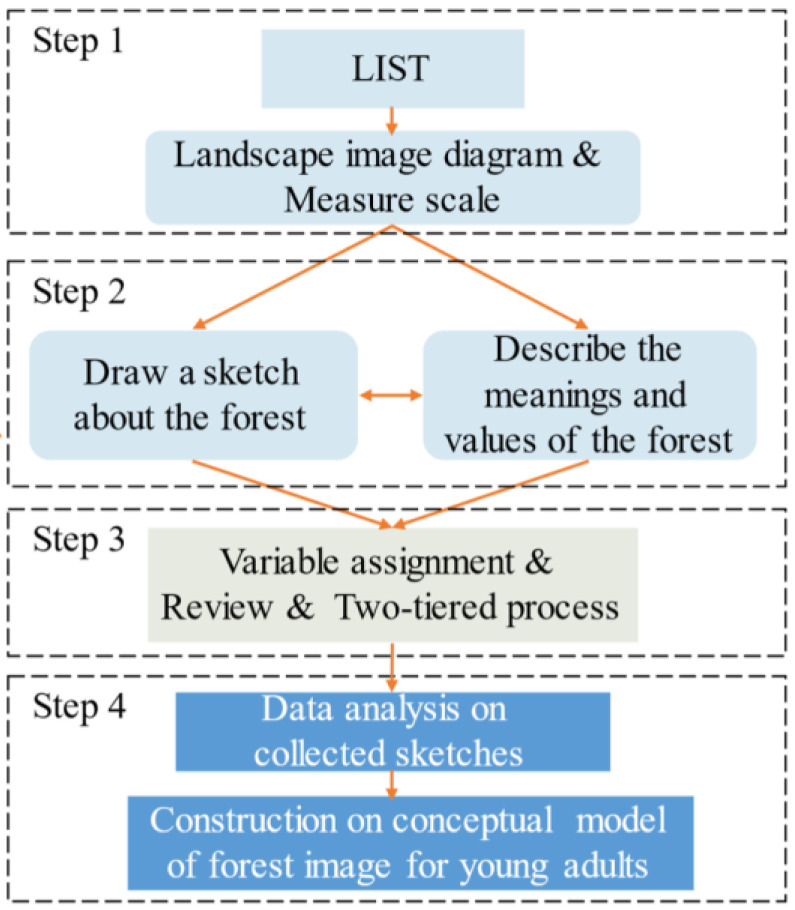
Main steps used to construct a conceptual model of forest-landscape image for young adults.

Four steps were taken to develop a conceptual-image model of the interaction between the young adults and the forest in Changsha, Central China (see Figure 1). Firstly, a diagram of the landscape-image-sketching technique was constructed. Secondly, the respondent-perception measurement scales for forest landscapes were developed. Thirdly, data on landscape-image sketches were analyzed, according to the respondent-perception measurement scale. Finally, the conceptual-forest-image model for young adults was constructed.

### 2.1. The Study Area

**Figure 2:**
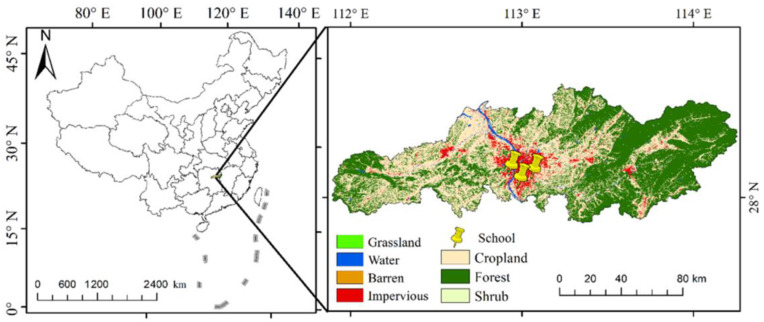
The study area.

Changsha is the capital city of Hunan province, Central China, in the range of 111°53′ to 114°15′ N and 27°51′ to 28°41′ E (see Figure 2). It is a pilot area of the national ‘resource-saving and environment-friendly society’ construction and was awarded the National Forest City in 2006. In addition, it has a population of 10.24 million and land area of 11,819 km^2^, which was dominated by the subtropical evergreen broad-leaved forest, with 59.82% of forest coverage rate in 2021. Changsha is also a famous tourism destination, with lots of natural and cultural heritage sites in Central China.

### 2.2. Landscape-Image-Sketching Technique

The landscape image was a result of the observers’ subjective understanding of the information which they received from the landscape object, for example, the individual characteristic and meaning and the morphological structure [31]. Ueda et al. (2012) stated that the landscape image was generated from the interactional effect between the observer and the landscape where he/she was [3]. According to the previous studies, the forest-landscape image includes spatial view, linguistic knowledge, self-orientation, and social meaning of the forest landscape [32,33,34,35]. The formation of forest-landscape images is a complex process, in which the observers’ consciousness, perception, attitude, and social normalization interact with the aforementioned elements [34]. More information about the forest-landscape diagram can be found by referring to Ueda et al. (2012) and Wang and Luo (2022).

Linguistic knowledge usually concerns the use of oral or written words to depict landscape elements. Previous studies showed that linguistic knowledge was considered as the first step for the respondent to classify forest-landscape elements in the sketches which contained landscape elements and forest types [34,35]. Spatial view refers to the viewing angle of the observer of the forest, which usually includes the close-up view, the sideways view, the bird’s-eye view, and the distant view. Self-orientation of the observers is represented by the relationship between various landscape elements and the stand-point of the observers in the sketches, which usually contains the single object, the objective landscape, the surrounding place, and the scenic place, and so on. Social meaning could be reflected by the interpretation of the respondents’ oral language, the landscape elements, and the self-orientation in the sketches. Essentially, the forest landscape not only reflects the individual interest of the respondent, but also implicates the specific social meaning endowed on the forest by the respondent. The social meaning could be divided into eight forms; for instance, the scenic view, the forest structure, the recreational space, the forestry operation, ecological system, natural resources, the living world, and the symbolic place.

The measurement scale of the forest-landscape image for respondents refers to Wang and Luo (2022).

### 2.3. Data Collection and Processing

From April to May 2018, this research used convenience sampling to select 140 young adults (18–25 years old) who had lived in Changsha, Hunan Province for more than ten years. There was no universal standard for a “young adult”, so this research used the age interval between 18 and 25 years to define this group of “young adults”. Then, 140 copies of forest landscape-image sketches were collected on site in the Central South Forestry & Technology University, Hunan Women’s University, Hunan Normal University, of which 131 copies were valid (response rate of 94%). The research showed that female respondents accounted for 61% of all the interviewed respondents.

The main research process consisted of five steps. Firstly, a preliminary communication with the respondents was conducted to confirm the investigation target, process and requirements, and collect the respondents’ demographic data, including their age, gender, studying major, grade, permanent residence, duration of residence in Changsha, and so on. Secondly, the respondents were asked to answer the following question: “what firstly appears in your minds when listening to the word of ‘forest’?” Thirdly, the respondents were expected to finish a painting by themselves of the forest in their minds on A4 paper in less than 15 min. If the respondents could not draw some landscape elements, they were asked to make a circle with the name of the landscape element as a substitute for the image on the paper. Fourthly, the respondents were required to describe concisely what kinds of landscape elements were included and what social meanings were contained in their paintings. Finally, the collected paintings (including 31 elements) were analyzed in terms of linguistic knowledge, spatial view, self-orientation, and social meaning. This process resulted in 31 landscape elements, which were further analyzed using MS Excel, whereas “1” or “0” was marked for an existing or non-existing element, respectively.

## 3. Results

### 3.1. Linguistic Knowledge

The results showed that ‘herbaceous plants’ (64.12%) and ‘sky’ (60.31%) were the top two in the frequency of landscape elements in sketches, followed by ‘creatures’ (43.51%), ‘water’ (36.64%), ‘trails’ (25.95%), ‘terrain’ (34.35%) and ‘artificial object’ (30.53%). Meanwhile, ‘people’ and ‘brightness’ only accounted for 5.34% and 3.05%, respectively (Table 1). The results also revealed that the respondents preferred natural landscape elements, for example, mountain, sky, water, animal, and plant, implying that they love nature. In addition, the respondents preferred ‘sky’ rather than ‘brightness’ in the paintings, due to the perceived discrepancy in the interview of the former being outside of the forest and the later inside the forest. There existed a tendency for the respondents to prefer ‘herbaceous plants’, ‘terrain’, ‘creatures’, ‘water’ and ‘sky’ rather than human-made landscape elements, such as ‘artificial object’ and ‘trail’.

The type of the forests was preferred as follows: ‘broadleaf forest’, ‘mixed forest’, ‘needleleaf forest’, ‘bamboo forest’, ‘unknown type’, and ‘fallen trees’ (Table 1). The research stated that the respondents preferred ‘broadleaf forest’, which was most commonly visible around the living environment, alongside the road or in the park related to their daily life, study and recreational activities. In addition, it reflected the fact that the respondents preferred the local native vegetation around their living environment. The ‘mixed forest’ was not popular with the public, who did not have a clear understanding of the forest type. The ‘bamboo forest’ received limited notice, because lots of respondents did not regard it as one kind of a forest type, and neglected it when painting the forest. The results showed that the respondents’ perception of the forest could be affected by lots of factors, such as the place of residence, the restriction of their cognitive abilities, and the living environment. Finally, ‘fallen tree’ was paid the least attention by the respondents, which not only demonstrated the fact that the respondents did not enjoy the dead wood or fallen trees, but indicated that they walked in the forest less each day and did not show less concern for nature.

### 3.2. Spatial View

**Figure 3:**
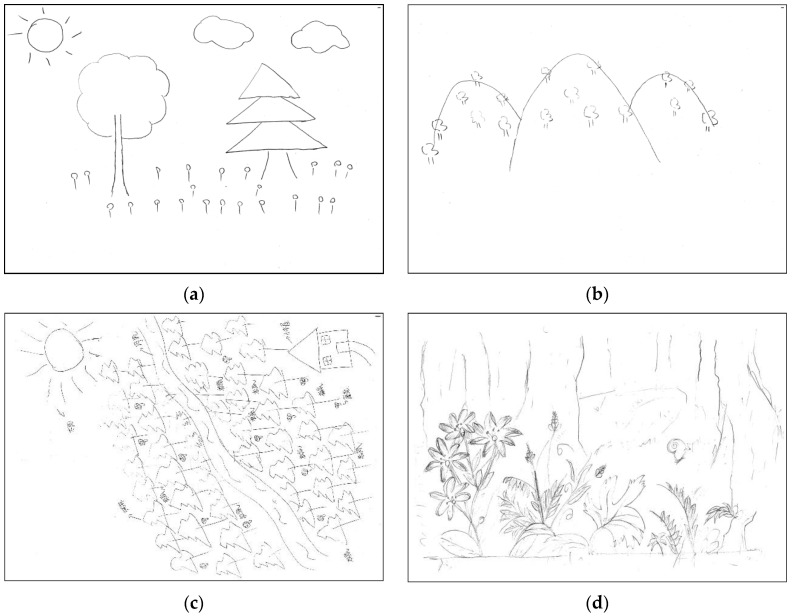
Classification of spatial views: (**a**) sideways view; (**b**) distant view; (**c**) bird’s-eye view; (**d**) close-up view.

The results demonstrated that ‘sideways view’ for the forest landscape was ranked top, accounting for 74.81% of the total spatial-viewing sketches (Table 2), with, for instance, the whole tree and the broadleaf tree (Figure 3a). A ‘distant view’ ranked second, taking up 31.30%, with, for instance, a tree with blurred crown (Figure 3b). A ‘bird’s-eye view’ and ‘close-up view’ were little noticed in the sketches, with 16.03% and 5.34%, respectively. The former was represented as a river with a clear boundary where a belt of pine-tree forests was painted low on the slopes of the hill (Figure 3c), and the latter was considered as the texture of the trunk and as the flower, which were exhibited clearly in the sketches (Figure 3d). In addition, it was noted that the total percentage did not equal one, as the spatial view of the sketch could be repeatedly calculated according to the landscape elements of different viewing points.

The results showed that the ‘sideways view’ dominated in the spatial view, with a few whole broadleaf or needleleaf trees. This was a similar phenomenon in daily life, where greenery was around houses or there were street trees on both sides of the road. When hearing the word “forest”, most of the respondents often did not imagine a picture of a lush forest, because they were more familiar with scattered tree species. It indicated that most respondents observed the forest at a medium distance, from the outside, and few looked at it from the inside, meaning that the breadth of the forest was neglected by the respondents.

### 3.3. Self-Orientation

**Figure 4:**
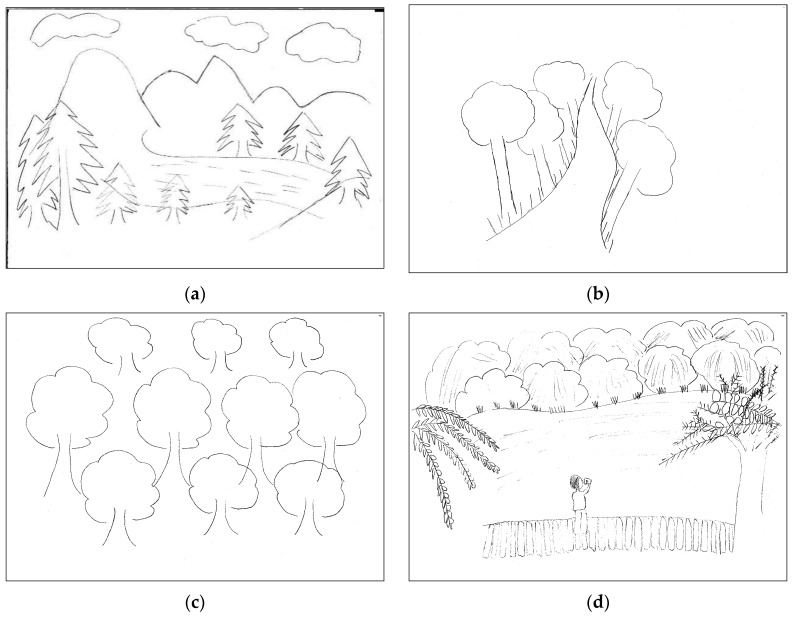
Classification of the self-orientation: (**a**) objective scene; (**b**) surrounding place; (**c**) single object; (**d**) scenic place.

The results showed that ‘objective scene’ ranked first (76.34%) (Table 2), representing natural landscape elements which dominated in the sketches, such as grasses, trees, rivers, and mountains (Figure 4a). A ‘surrounding place’ was second (17.56%), in which a road was painted in the center of a sketch with both sides having distinctive tree-trunks, indicating the respondent was walking on the road (Figure 4b). A ‘single object’ was in third place (5.34%), with, for instance, only a pattern of trees, which could be observed in the sketches (Figure 4c). A ‘scenic place’ was the lowest, accounting for only 3.82%, and usually showed the objective landscape elements, for example, grasses, trees, lakes, and bridges, and a person standing on the bridge and looking far away, to exhibit the respondent’s own viewpoint (Figure 4d).

### 3.4. Social Meaning

The results showed that ‘scenic view’ and ‘forest structure’ were preferred in the sketches, accounting for 27.48% and 27.48%, respectively. An ‘ecological system’, a ‘recreational space’, and ‘life world’ were often mentioned, accounting for 23.66%, 15.27% and 21.37%, respectively. The ‘natural resources’ was rather low, accounting for 5.34%. In addition, a ‘symbolic place’ and a ‘forestry operation’ were almost neglected in the sketches, with each only taking up 1.53% (Table 2).

**Figure 5:**
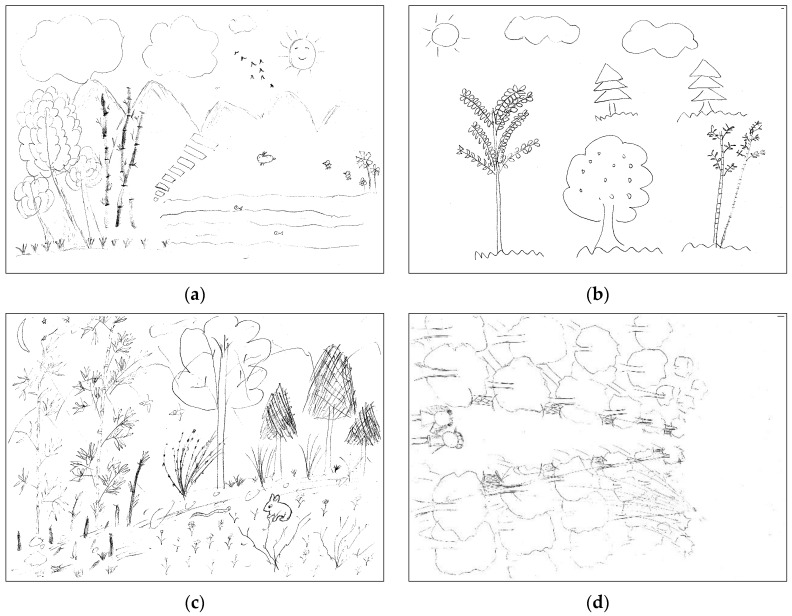

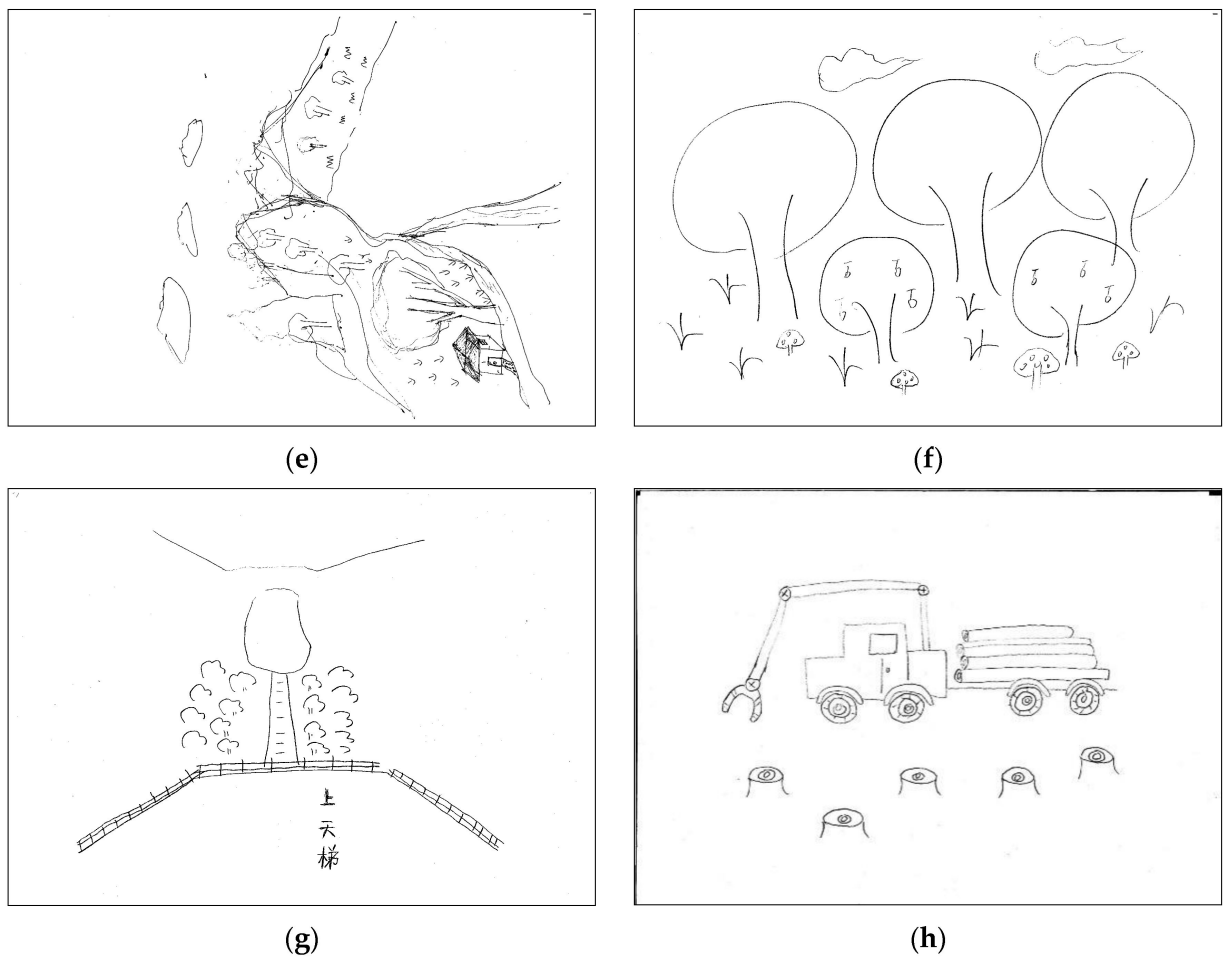
Classification of ‘social meaning’: (**a**) scenic view; (**b**) forest structure; (**c**) ecological system; (**d**) recreational space; (**e**) life world; (**f**) natural resources; (**g**) symbolic place; (**h**) forestry operation.

A ‘scenic view’, such as ‘objective scene’ or ‘scenic place’ depicted a beautiful forest sketch, such as the foreground and background from the ‘sideways view’ and ‘distant view’ (terrain), respectively (Figure 5a). ‘Forest structure’ was represented in the horizontal and vertical distribution of layers, including the trees, shrubs and ground covers in a sketch (Figure 5b). An ‘ecological system’ referred to an idealized objective scene, including a diversity of plants and animals, for example, grasses, trees, rabbits, birds, and snakes (Figure 5c). A ‘recreational space’ usually indicated a ‘surrounding place’ for the public recreational activities, in which the trail for a walk in the forest was painted in a sketch (Figure 5d). A ‘life world’ referred to the settlements around a forest, such as houses in the forest (Figure 5e). The ‘natural resources’ were generally exhibited by lots of non-timber resources on the ground inside a forest, for example, bamboo shoots, and mushrooms under some trees (Figure 5f). A ‘symbolic place’ was usually represented by single objective landscapes, for example, an ancient tree or a symbolic landscape in a sketch which usually left a deep impression on the viewers. In the sketches, the close-up view was often used to represent the symbolic one, in the case of Tianmen Cave in Tianmen Mountain National Forest Park, Hunan (Figure 5g). In addition, a ‘forestry operation’ referred to the operational method or processes of the forest industry, such as forest harvesting (Figure 5h).

The results also stated that when listening to the word “forest”, the respondents would imagine a symbol of a tree or trees, and other natural landscape elements, for example, lakes, streams, animals and non-timber resources, indicating that there was a combination of a diversity of landscape elements inside the forest in the respondents’ imagination, for example, a natural landscape cluster of trees, grasses, and fields. Interestingly, water was the favorite to be used in the sketches frequently. This imagination resulted in the frequent appearance of ‘forest structure’ and ‘ecological system’ in the sketches. The respondents preferred to paint mountains as backgrounds in a beautiful rural forest landscape as a favorite ‘scenic view’ in the sketches. Interestingly, there was a house under a chimney in the sketch, which indicated that the respondents unconsciously regarded the rural landscape and the fragmented urban green spaces closely connected to the residential place as part of the forest. It was vital to present a life world in the sketches. In addition, the respondents initially imaged the primary forest rather than the forest scenic areas when hearing the word “forest”, due to the limited time spent in the forest or having less impression of the forest landscape in their minds. There were, however, a small number of the symbolic places in sketches. The rise of recreational space accorded with the occurrence of the trail, resulting in only taking a walk instead of other recreational activities in the forest. This phenomenon reflected the fact that some of respondents undervalued the recreational values of the forest, on account of the shortage of positive experiences in the past or the difficulty of acquiring interesting experiences when in contact with nature. Natural resources were less depicted in the sketches because the respondents usually seldom walked into the forest to pick up edible fungi and berries. In addition, the forestry operation was seldom referred to, because of less familiarity with forestry on the part of the respondents since the ban on logging at the end of the 1990s.

## 4. Discussion

The results demonstrated that there existed a fundamental differences in ways of viewing the forest through individual perceptions, rather than normative views on the forest [3], depending, for example, on the observer demographic, geographical region, human activities, and cultural context [18,36,37]. This study also supported the fact that there was a significant difference between the subjective perception of forests and the objective forest landscape in China, representing the typical landscape image of a forest as a subjective and practical place for the respondents. Furthermore, there was a house under a jerkinhead roof or chimney in the forest-landscape image in the Chinese context, which stood for the idyllic scenery around the “home” in the form of a kind of virtual understanding and perception. In addition, this study proved that the landscape-image-sketching technique could be successfully applied in Eastern and Western countries to research individual perception of forests [21].

From the point of view of linguistic knowledge, ‘broadleaf forest’, as the type of the native vegetation in Central China, was a favorite type of forest in this study. Similarly, ‘mixed forest’ and ‘broadleaf forest’ were preferred in Russia and Japan, respectively [6,20]. The previous studies showed the preference was affected by lots of causes, for example, cultural and geographical difference [38], the living environment, familiarity [39], landscape dependency [40], and the high or low frequency of visiting the forest [26], and so on. For instance, the respondents did not like the ‘fallen tree’ in China, while the Japanese did. Previous studies showed that the higher the level of forestry education received by someone, the stronger the willingness to be close to nature [26]. Currently, natural education of the forest is gradually becoming valued in society, in which there has been a serious shortage of variety of content, curricula, and tutors for national education in China [2].

From the point of view of landscape elements, the respondents preferred the forest as a ‘natural space’ in China for painting natural landscape elements, such as ‘herbaceous plants’ and ‘sky’. Interestingly, previous studies demonstrated that the Japanese preferred to paint human landscape elements in sketches, such as, ‘trails’, ‘people’, and ‘brightness’ [41], due to a ‘recreational space’ being perceived in Japan [26]. In addition, the brightness in the sketches was noticed, and indicated that the respondent deeply understood the change of landscape elements in a forest by establishing a close connection between himself or herself and the forest, with daily visits to the forest [42].

For the spatial view, the respondents preferred a ‘sideways view’ to observe the forest, indicating that the observers were a little far from the forest, with poor accessibility to the forest. Previous studies also demonstrated that respondents from Japan preferred the ‘bird’s eye view’ and ‘close-up view’, while those from Irkutsk and the Kamchatka Peninsula of Russia preferred the ‘bird’s eye view’, and those from Moscow in Russia the ‘sideways view’ and ‘close-up view’ [3]. Previous studies also stated that the distance from the forest, the accessibility to the forest [43], and the terrain of the forest area [8] were important factors in the observers’ forest spatial cognition. Before 1990s, most people lived in the rural area and went to the forest daily to pick living resources, such as mushrooms and branches. However, with the development of modern technology and the accelerating urbanization, people gradually moved to cities and had fewer visits to the forest, because they were far away from the forest and got few living resources from the forest. In addition, the results showed that the respondents usually lived in hilly terrain in Central China.

In the self-orientation and ‘social meaning’, the objective scene’ and ‘scenic view’ of the rural landscape played a leading role in the sketches, which indicates that the respondents considered the forest most as an objective forest, not a subjective on, and did not make efforts to push the interaction between themselves and the forest. The urbanization rate, traditional landscape sketches, and the perception of forest value attributes were considered as the important factors in self-orientation and social meaning. With the increase of the urbanization rate from 30.89% to 64.72% from 1999 to 2021 in China, the Chinese people felt homesick for their own homes around the forest in the rural area and were living in cities for a long time, making them unconsciously paint houses under chimneys in a rural scene. This traditional style of landscape sketches in China was characteristic of the combination of foreground and background in a sketch to depict beautiful rural landscapes, with various viewing points [20]. In addition, the public emphasized the natural attributes of the forest for timber production rather than the social ones for the provision of ecological products, within the concept of the traditional cognition of the forest [2]. Previous studies showed that the respondents in Russia preferred the natural resources and considered the forest as an important space for traditional-living resources, so that they used mushrooms and berries as featured landscape elements in the sketches; instead, respondents in Japan thought of the forest as the ‘surrounding space’ and ‘recreational space’, due to their frequent recreational and experience activities in the forest, with good accessibility to the forest [3].

**Figure 6:**
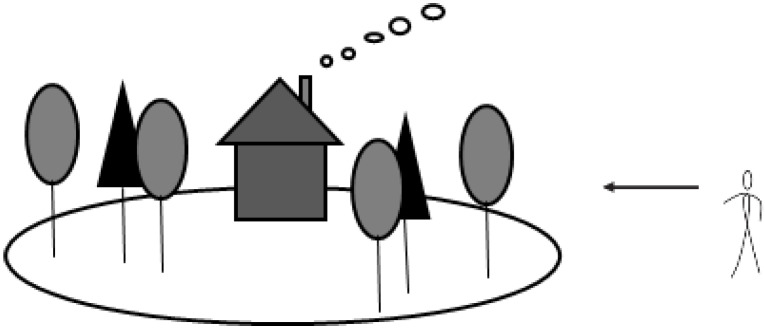
Conceptual model of forest-landscape image for young adults, Central China.

Therefore, with the statistic and content analysis of linguistic knowledge, spatial view, self-orientation, and social meaning for young adults in Central China, this manuscript tentatively establishes the conceptual model of forest-landscape image for young adults in Central China, as shown in Figure 6. In China, the typical landscape image of a forest was a subjective, practical place. Mixed forest and houses were perceived from the outside of forests, from a middle distance. The forest was only a backdrop for people’s living surrounding and the rural scenery around their own homes, instead of the objective forest in their minds.

## 5. Conclusions and Implications

This paper analyzed individual perceptions of the forest-landscape image using the landscape-image-sketching technique and constructed a conceptual model of landscape image for the forest in a Chinese context. The findings showed that the forest was regarded as the people’s living surroundings and the rural scenery around their own homes, instead of the objective forest, in their minds. Linguistic knowledge of the forest landscape, including broadleaf forest, objective scene, sky, artificial view, and scenic view depicted the forest as a pleasant landscape. The forest was also considered as an important habitat and a limited space to meet the needs of the people’s living, production, and recreation. The respondents therefore chose to appreciate the beauty of the forest from a sideways view, and neglected the cluster of landscape elements inside the forest and the breadth of the forest. Natural values such as, ‘forest structure’, ‘ecological system’ and ‘scenic view’, dominated in the social meaning in the sketches.

In practice, it is important to increase public or elective courses about the forest or nature, to enhance the public’s awareness of the objective forest, especially for children and teenagers; the courses should be about the knowledge of forest structure, forestry operations, inter-forest attractions, and so on. With the construction of a natural-reserve system with national parks as the main body of this in China, a variety of experiences in these natural reserves would be developed for the public, and given priority in the future. Our findings showed that many university students still did not understand what the difference between the natural-reserve system and national parks was.

Future research is needed to verify the conceptual model of the forest landscape image for young adults on a broader scale and to explore the construction of the model of the forest-landscape image for other age groups. In addition, we also found that the forest could be considered as a subjective forest in Japan [8], and an objective one in Europe [3]. It was necessary to analyze the factors of a subjective or objective forest perceived by the public, such as the change among generations [44], and the intergenerational difference in education [45], place attachment [40], and recreational experience [7]. Meanwhile, the new emerging techniques, for instance eye tracking and brainwave measurement, are being widely used in landscape cognition.

In addition, there were some limitations in this manuscript. Firstly, limited samples were collected, and the respondents, who lived in Changsha, could not represent all young adults in Central China. Secondly, some respondents were good at drawing the sketch by hand, which might insufficiently exhibit some landscape elements in the sketch.

## Figures and Tables

**Table 1:** Statistical data for landscape element and forest types in sketches.

Classification	Sub-Classification	Percentage
Natural landscape	Herbaceous plants	64.12%
	Terrain	34.35%
	Creatures	43.51%
	Water	36.64%
	Brightness	3.05%
	Sky	60.31%
Human landscape	Trails	25.95%
	Artificial objects	30.53%
	People	5.34%
Forest types	Mixed forest	25.95%
	Needleleaf forest	9.92%
	Broadleaf forest	57.25%
	Bamboo forest	4.58%
	Unknown	7.63%
	Fallen trees	1.53%

**Table 2:** Statistical data for spatial view, self-orientation, and social meaning in sketches.

Classification	Sub-Classification	Percentage
Spatial view	Close-up view	5.34%
	Sideways view	74.81%
	Bird’s-eye view	16.03%
	Distant view	31.30%
Self-orientation	Single object	3.82%
	Objective scene	76.34%
	Surrounding place	17.56%
	Scenic place	3.82%
Social meaning	Forest structure	27.48%
	Scenic view	27.48%
	Recreational space	15.27%
	Symbolic place	1.53%
	Ecological system	23.66%
	Natural resources	5.34%
	Forestry operation	1.53%
	Lifeworld	21.37%

## Data Availability

The data presented in this study are available in the Supplementary Materials.

## Supplementary Materials

The following supporting information can be downloaded at: https://www.mdpi.com/article/10.3390/ijerph20042986/s1, Table S1: The landscape images data.
